# Investigation of Second- and Third-Harmonic Generation in Few-Layer Gallium Selenide by Multiphoton Microscopy

**DOI:** 10.1038/srep10334

**Published:** 2015-05-19

**Authors:** Lasse Karvonen, Antti Säynätjoki, Soroush Mehravar, Raul D. Rodriguez, Susanne Hartmann, Dietrich R. T. Zahn, Seppo Honkanen, Robert A. Norwood, N. Peyghambarian, Khanh Kieu, Harri Lipsanen, Juha Riikonen

**Affiliations:** 1 Aalto University, Department of Micro and Nanosciences, Tietotie 3, FI-02150 Espoo, Finland; 2 University of Arizona, College of Optical Sciences, 1630 E University Blvd, Tucson, AZ 85721, USA; 3 Technische Universität Chemnitz, Semiconductor Physics, Chemnitz 09107, Germany; 4 University of Eastern Finland, Institute of Photonics, P.O. Box 111, FI-80101 Joensuu, Finland

## Abstract

Gallium selenide (GaSe) is a layered semiconductor and a well-known nonlinear optical crystal. The discovery of graphene has created a new vast research field focusing on two-dimensional materials. We report on the nonlinear optical properties of few-layer GaSe using multiphoton microscopy. Both second- and third-harmonic generation from few-layer GaSe flakes were observed. Unexpectedly, even the peak at the wavelength of 390 nm, corresponding to the fourth-harmonic generation or the sum frequency generation from third-harmonic generation and pump light, was detected during the spectral measurements in thin GaSe flakes.

Since the discovery of graphene,^1^ layered materials have gained interest around the world. Graphene has interesting optical properties which have been exploited in different applications, including as a saturable absorber in pulsed fiber lasers,^2^ in optical modulators^3^,^4^ and in devices based on third-order nonlinear optics to generate optical bistability, regenerative oscillations and four-wave mixing^5^. Recent studies have shown that graphene detectors can operate over a very broad bandwidth due to the zero band-gap of graphene^6^,^7^,^8^. However, the zero band-gap of graphene also gives rise to a high optical absorption coefficient, which is detrimental in guided-wave devices. This gives further motivation to study the optical properties of other two-dimensional semiconductor materials with a non-zero bandgap. Among those, the photoluminescence (PL) properties of the monolayer transition metal dichalcogenides such as MoS_2_, MoSe_2_ and WSe_2_ are rather well studied^9^,^10^,^11^. However, there are only few studies on gallium chalcogenides. Recently, the use of single- and few-layer GaSe in transistor and photodetector applications has been reported^12^,^13^,^14^,^15^,^16^. For layered gallium selenide, PL has been observed in the wavelength range around 620 nm–640 nm^17^. High anisotropy in the Raman spectra and PL of GaSe flakes was recently reported showing the rich physics at the mesoscale with respect to the bulk phase^18^. Bulk gallium selenide is also a well-known material for nonlinear optics and has been used for second-harmonic generation (SHG) at near- and mid-infrared (IR) wavelengths^19^,^20^,^21^. Third-order nonlinearities are the basis of all-optical devices employing self-phase modulation and four-wave mixing applicable to telecommunications. The demand for miniaturized devices motivates the assessment of the nonlinear optical properties of nano-scaled layered materials at the predominant telecommunications wavelength (around 1550 nm). In recent years, the second- and third-order optical nonlinearities in layered materials (including graphene, MoS_2_, WS_2_ and h-BN) have gained great interest^22^,^23^,^24^,^25^,^26^,^27^,^28^. After the submission of our work, a study of layer-dependent second-harmonic generation in few-layer gallium selenide at excitation wavelengths from 800 nm to 1080 nm was also reported^29^.

In this paper, we report on employing multiphoton microscopy with a compact 1560 nm femtosecond fiber laser to study the nonlinear optical properties of few-layer GaSe flakes. We observed that the second- and third-order nonlinearities are very sensitive to the number of layers. We also discovered the generated light at the peak wavelength of 390 nm from ~40 nm thick GaSe. To our knowledge, this is the first systematic optical nonlinearity study of few-layer GaSe using the excitation wavelength around 1560 nm of relevance for telecommunication applications.

## Results and discussion

### Optical microscopy

**Figure 1:**
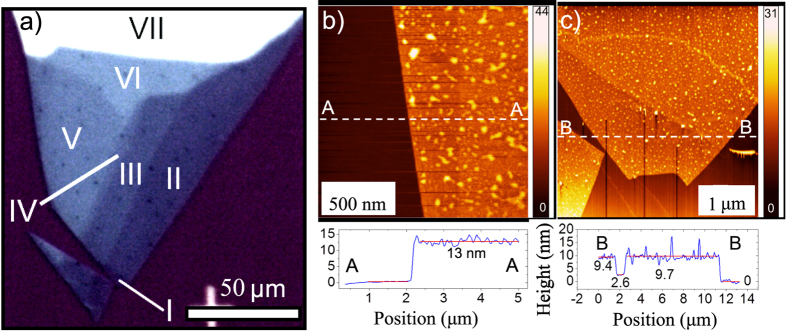
**a**) Optical image of a mechanically exfoliated GaSe flake. Areas with different numbers of layers are marked with Roman numbers from I to VII. These Roman numbers do not directly correspond to the number of layers. AFM micrographs of the few-layer GaSe flake: **b**) from the left edge of the flake and **c**) from the tip of the flake. The cross-sectional plots below the AFM images are taken from the white dashed lines.

Figure. 1a presents an optical microscope image of a GaSe flake. The optical contrast corresponds to a different number of layers labeled by Roman numbers I to VII.

### Surface morphology

The few-layer GaSe flake shown in Fig. 1a was characterized using contact-mode atomic force microscopy to determine the number of layers in the different areas. Since the thickness of single-layer GaSe is known to be ~0.98 nm,^12^,^14^ the flake thickness in nm corresponds roughly to the number of layers. The AFM micrograph from the left edge of the GaSe flake is presented in Fig. 1b. The height of the area V (in Fig. 1a) from the substrate is found to be 13 nm indicating 13 layers. An AFM micrograph from the tip of the flake (Fig. 1c) shows two different areas with a different height. The thinnest area (area I in Fig. 1a) contains 7 layers of GaSe, estimated from the cross-section (9.7–2.6 = 7.1 nm). The additional 2.6 nm on the left side of the SiO_2_ substrate comes from an artifact due to the background subtraction process (flattening). From the same cross-section, the thickness of area II corresponds to 9 layers. The thickness differences between the adjacent areas II and III in Fig. 1a was estimated to be 2 nm and the areas III and IV to be 1 nm. Therefore, areas III and IV are 11 and 12 layers thick, respectively. The area VI was estimated to contain 14 layers. The estimated thicknesses are listed in Table 1. The accuracy of the thickness determination is ±1 layer. Area VII was measured to contain ~40 layers. 10–20 nm high dots in the AFM micrographs are assumed to be oxidized GaSe, mainly occurring on the crystal defects^30^.

### Multiphoton imaging

**Figure 2:**
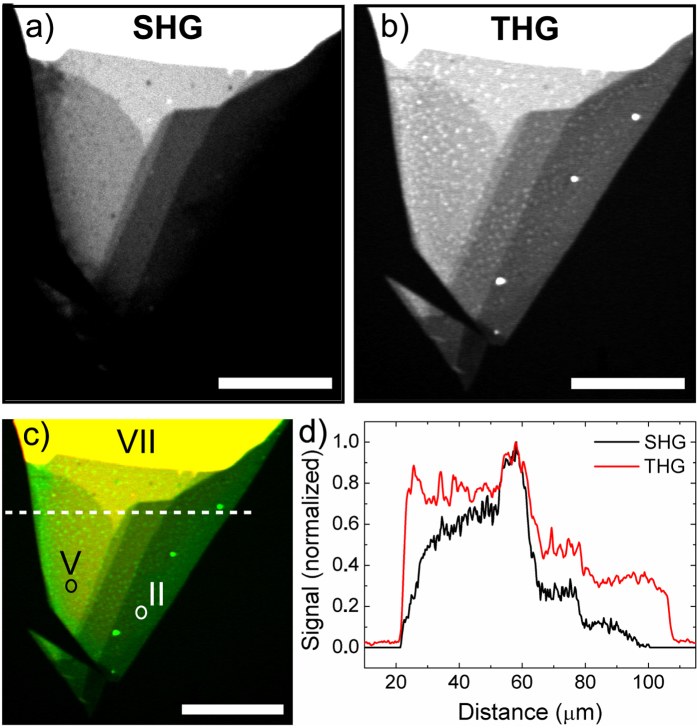
**a**) SHG and **b**) THG images of the few-layer GaSe flake, **c**) RGB composite image generated from the SHG and THG images, and **d**) cross-sections of the SHG and THG signals taken from the white dashed line in **c**). The spectra of the generated light have been measured from the points marked with Roman numbers II, V and VII in **c**). Scale bars are 50 μm.

SHG and third-harmonic generation (THG) images of the GaSe flake were recorded simultaneously and are shown in Fig. 2a,2b, respectively. Although the SHG signal was much stronger than the THG signal, the image contrast was scaled in order to visualize most layers in the images. The areas with different number of layers are clearly distinguishable in both SHG and THG images and the signal intensity increases as the number of layers increases. The THG image gives higher contrast between the substrate and the GaSe flakes. The SHG signal is observed to decrease near the edges while the THG signal is constant over the area with the same number of layers. Yin *et al.* observed strong edge effects for SHG from single-layer MoS_2_.^31^ They observed that the edge effect is strongly dependent on the excitation wavelength. It was recently showed that GaSe flakes also display high anisotropy in the optical properties at the crystal edges^18^. In our case, the photon energy of the SHG is below the band-gap of GaSe (~2.1 eV)^32^ and the SHG signal decreases significantly near the edges of the flake. Fig. 2c shows an RGB composite SHG-THG micrograph obtained by combining images 2a and 2b. The SHG and THG signals are in the red and green channels of the RGB image, respectively. The areas that simultaneously show a high intensity for both SHG and THG appear yellow in the composite image. The cross-sectional profiles of SHG and THG signals from the dashed line in Fig. 2c are plotted in Fig. 2d. The quenching of the SHG signal near the edges is clearly visible in the profile.

**Figure 3:**
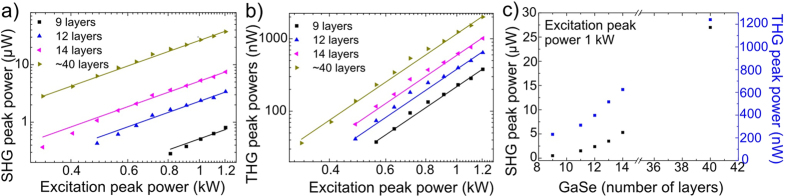
Power dependence of the **a**) SHG and **b**) THG signals. **c**) SHG and THG signals as a function of the number of the GaSe layers measured with 1 kW excitation peak power.

The excitation light power dependences of the SHG and THG signals on a logarithmic scale for different layer thicknesses are presented in Fig. 3a,b, respectively. The lines are fits to square (a) and cubic (b) power dependences. The measured SHG and THG signals follow the square and cubic power dependences quite well proving that the signals are SHG and THG. A power dependence measurement was realized by adjusting the laser intensity producing a peak power from 0.3 kW to 1.2 kW. The SHG and THG signals for different layer thicknesses were found to be roughly independent of the excitation peak power. In Fig. 3c, the SHG and THG signals for 1 kW excitation peak power are plotted as a function of the number of GaSe layers. These results show that the SHG signal is proportional to the flake thickness. The highest SHG peak power was about 28 μW for an excitation peak power of 1 kW. However, the flake is extremely thin compared to typical crystals used in frequency doubling. The highest THG peak power was obtained for the ~40 nm thick flake. The coherence length for backward generated THG can be calculated from 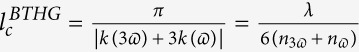. With the excitation wavelength λ = 1560 nm, we used refractive indices of n_ω_ = 2.75 (Ref. 33) and n_3ω_ = 3.19 (measured by ellipsometry at the wavelength of 516 nm) for fundamental and THG light, respectively. The estimated 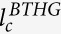 is about 45 nm. For SHG, 
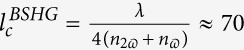
 nm using *n*_2*ϖ*_ = 2.86 (taken from Ref. 33 at the wavelength of 800 nm). For both SHG and THG, the coherence length – even for the backward generated SHG and THG – is longer than the thickness of the flake. Below the coherence length, harmonic signals should increase when the thickness increases and our measurements are consistent with this theory. The surface second-order nonlinear susceptibility can be estimated using the equation^28^


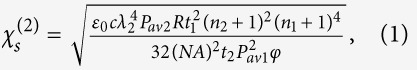


where *R* is the repetition rate (75 MHz), *t*_*i*_ is the pulse width (251 fs), *P*_*avi*_ is the average power (i = 1: excitation, 2:SHG), *n*_*i*_ is the refractive index of the substrate material (i = 1:excitation [1.45], 2:SHG [1.44]), NA is the numerical aperture of the objective (0.5) and 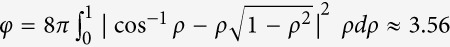. The effective bulk-like second-order susceptibility is estimated taking the material thickness 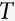 into account by 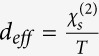. The estimated *d*_*eff*_ for different GaSe thicknesses are presented in Table 2. The highest estimated value is 18 pm/V. The second-order nonlinearity coefficient of d_22_ = 53 pm/V has been earlier reported for the bulk GaSe at the excitation wavelength of 10 μm^34^.

The third-order optical nonlinearity coefficient 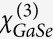 was estimated to be around 1.2 ± 0.2*10^–8^ esu using single-layer graphene as a reference (
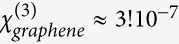
esu^35^). The estimation was done with the equation 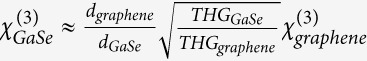, where *d*_*graphene*_ is 0.3 nm, and *THG*_*graphene*_ and *THG*_*GaSe*_ were measured using the same excitation power. The estimated χ^(3)^ values for different thicknesses are shown in Table 2. The χ^(3)^ of graphene is known to be exceptionally high due to the resonant condition for the excitation and THG signals^36^. The absorption of the THG signal (THG photon energy of 2.38 eV is higher than the band-gap of GaSe (~2 eV)) has not been taken into account and therefore, the χ^(3)^ of GaSe could be even higher than estimated.

### Spectra of generated light

**Figure 4:**
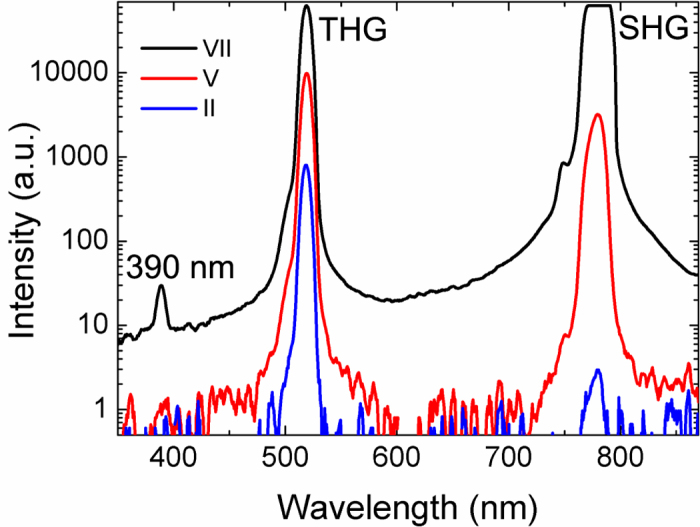
Measured spectra of the generated light from three different positions (different thicknesses) on the flake. The positions are marked by Roman numbers II, V and VII in Fig. 2c.

The spectra of the generated light measured from the three different areas (marked with circles and Roman numbers in Fig. 2c) are presented in Fig. 4. Strong peaks at the wavelengths of ~520 nm and ~780 nm were observed for the flakes with ~40 layers (area VII) and 12 layers (area V). The asymmetric line shape of the peaks is due to the spectrum of the excitation laser (the spectrum of the laser is shown in Methods). This asymmetry is clearly visible for the ~40-layer flake (area VII). The spectra confirm that the signals detected are second and third harmonics. From the area of 9 layers (area II), only the peak around ~520 nm was observed. This is due to the weaker SHG signal in this area and partly due to the reduction of the SHG signal near the edge as noted above (the measurement point is located exactly at the area where the SHG signal starts to decrease). The sensitivity of the spectrometer is also lower than that of the PMT used in multiphoton imaging. However, the measured spectra confirm that generated light consists of the SHG and THG signals and the contribution of the multiphoton excited photoluminescence is minimal. Even the peak at 390 nm can be clearly observed from the ~40-layer GaSe. This peak is exactly at the fourth-harmonic wavelength but it could also be the sum frequency generation from THG and pump light.

### Raman microscopy

**Figure 5:**
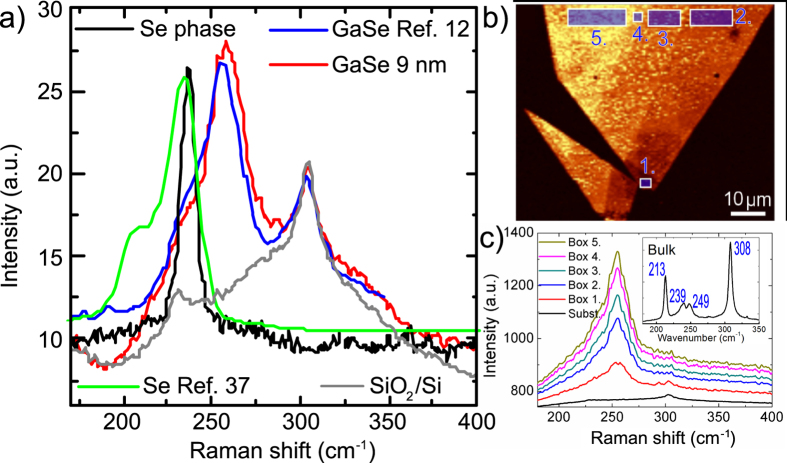
**a**) Raman spectra of 9-layer GaSe (red curve), single-layer GaSe (blue curve, adapted from Ref. 12), Se nanowire (black curve), Se (green curve, adapted from Ref. 37), and SiO_2_/Si substrate. **b**) Sum signal Raman map of the few-layer GaSe flake (wavenumber range from 235 to 275 cm^−1^), and **c**) the average Raman spectra of the different areas marked with numbered boxes in **b**). The Raman spectrum of bulk GaSe is shown as an inset in **c**).

The Raman spectra of the GaSe samples were also measured for verifying that GaSe does not decompose in the nonlinear microscopy experiments. The relatively high irradiance used during the nonlinear optical microscopy may induce modifications in the GaSe flakes resulting in spectral differences between the bulk and the thin layers. However, a comparison of our Raman spectrum using a 9 layer-flake with the Raman spectrum from a thin GaSe layer (1 nm) adapted from Ref. 12 shows clear similarities with GaSe, see Fig. 5a. Moreover, decomposition of GaSe was ruled out by performing a control experiment on a Se nanowire obtained by chemical decomposition of GaSe. The Raman spectrum obtained from a natural Se crystal is also shown^37^. These data confirm the chemical and structural stability of the GaSe thin layer flakes upon multiphoton microscopy.

In thin layers the Raman spectrum is dominated by the band centered around 255 cm^–1^. Raman imaging shows that this mode is common all around the flake although its intensity changes with the GaSe thickness as shown in Fig. 5b. The dependence of the Raman spectrum on layer thickness is illustrated in Fig. 5c where the spectra were averaged at different regions indicated by rectangular boxes in Fig. 5b. The spectrum from bulk GaSe is shown in the inset of Fig. 5c. By comparison with few-layer GaSe, we found a drastic difference with its bulk counterpart, which exhibits sharper bands around 213 and 308 cm^–1^. The reasons behind such a striking difference are still under investigation.

The band observed around 305 cm^–1^ cannot be attributed to the 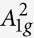 mode of GaSe observed in the bulk at 308 cm^–1^. This band clearly corresponds to SiO_2_/Si as shown by the spectrum obtained from the bare substrate. This is in contrast to the conclusion from Late *et al.* who attributes that band to the 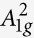 of GaSe and the low-frequency shift when decreasing layer thickness^12^.

## Conclusions

In conclusion, the optical nonlinearities of GaSe flakes with different number of layers were investigated using multiphoton microscopy with the excitation wavelength of 1560 nm, relevant for telecommunications. Both second- and third-harmonic generations were observed in few-layer GaSe flakes. The SHG and THG signals showed clear contrast for different numbers of GaSe layers. Second- and third-harmonic generation was confirmed by spectral analysis and also the peak at the wavelength of 390 nm was observed corresponding to the wavelength of fourth-harmonic generation or the sum frequency generation from THG and pump light. Strong nonlinear characteristics reported here for few-layer flakes demonstrate that GaSe is an intriguing material for all-optical signal processing, even as few atomic layer films. These results contribute to the development of GaSe thin-layer flakes for next generation nonlinear applications satisfying the continuous need for device miniaturization.

## Methods

### Sample preparation

GaSe flakes were grown by the Bridgman method. Few-layer flakes of GaSe were then obtained by mechanical cleaving from a bulk single crystal and deposited by gently pressing against the oxidized silicon substrate. In order to achieve maximal optical contrast the oxide thickness was 286 nm. Few-layer GaSe flakes were identified using optical microscopy and the number of layers was determined by atomic force microscopy (AFM) in contact mode.

### Multiphoton microscope

**Figure 6:**
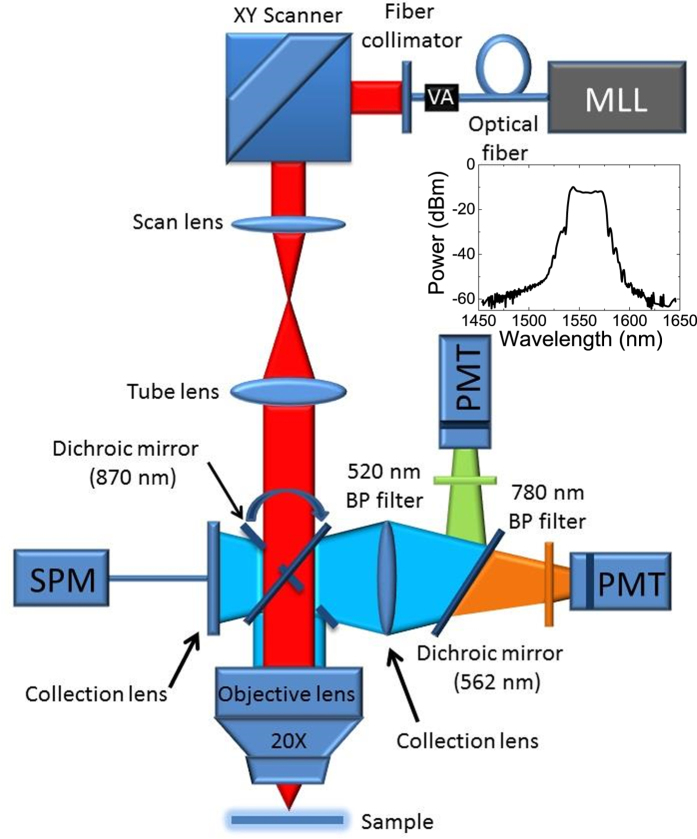
Schematic diagram of the multiphoton microscope. The inset shows the spectrum of the laser. MLL refers to mode-locked fiber laser, VA variable attenuator, SPM spectrometer, PMT photomultiplier tube and BP band-pass filter.

The nonlinear optical properties of the prepared GaSe flakes were investigated using a unique multiphoton microscope, built in-house, which is shown schematically in Fig. 6. The design of the microscope and the experimental procedure were previously reported^35^,^38^. The light source in the system is an amplified erbium-doped mode-locked fiber laser operating at a central wavelength of 1560 nm. The spectrum of the laser is shown in the inset in Fig. 6. The maximum average power of the laser is 60 mW with a repetition rate of ~50 MHz and ~150 fs pulse duration at the sample surface. The pulse peak power is estimated to be ~8 kW and the pulse energy is 1.2 nJ. The laser beam is scanned with a 2D galvo mirror system and focused on the sample using a 20x microscope objective. The measured focal spot size is ~1.8 μm. The backscattered second- and third-harmonic signals generated from each point on the sample are split into two paths using a long-pass dichroic mirror (cut-off at 562 nm) and then detected using photomultiplier tubes (PMT). Narrow band-pass filters are used to select SHG and third-harmonic generated signals at central wavelengths of 780 nm and 520 nm, respectively. The acquisition of the two channels is simultaneous, making the measurement conditions for both channels exactly identical regardless of any perturbations (external vibrations or fluctuations in laser power).

## Author Contributions

L.K. performed the optical, nonlinear optical, AFM and Raman characterization, analysed the data and completed the writing of the paper. A.S. contributed to the analyzing the data and writing the paper. S.M. built the nonlinear microscope and contributed to the nonlinear characterization. K.K. designed the multiphoton microscope, built the laser source and contributed to the building of the multiphoton microscope and the data analysis. S.Ha. and R.D.R. performed fabrication of the GaSe samples. R.D.R contributed also to the Raman characterization and analysis as well as the AFM analysis. J.R. and A.S. proposed the idea of the nonlinear characterization of few-layer GaSe. D.R.T.Z., S.Ho., R.A.N., N.P. and H.L. supervised the study. All the authors commented the paper.

## Additional Information

**How to cite this article**: Karvonen, L. *et al.* Investigation of Second- and Third-harmonic generation in Few-Layer Gallium Selenide by Multiphoton Microscopy. *Sci. Rep.* **5**, 10334; doi: 10.1038/srep10334 (2015).

## Figures and Tables

**Table 1:** The estimated number of layers on the flake.

**Area in** Fig. 1a	**Number of layers**
I	7
II	9
III	11
IV	12
V	13
VI	14
VII	40

**Table 2:** The estimated *d*_*eff*_ and *χ* ^(3)^ for the different numbers of layers.

**Number of layers**	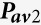 **(in W) when** 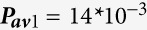 **W**	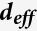	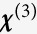
9	9.3*10^−12^	9.3 pm/V	1.15*10^−8^ esu
11	26*10^−12^	12.7 pm/V	1.17*10^−8^ esu
12	39*10^−12^	14.3 pm/V	1.16*10^−8^ esu
13	57*10^−12^	16.0 pm/V	1.18*10^−8^ esu
14	86*10^−12^	18.2 pm/V	1.22*10^−8^ esu

The measured average powers of SHG, *P*_*av*2_, are used to estimate the *d*_*eff*_.
